# Patient demographics, medical factors, treatment modalities and satisfaction at five traditional Chinese medicine practices in Switzerland: A cross-sectional study

**DOI:** 10.1371/journal.pone.0332586

**Published:** 2025-09-19

**Authors:** Yingchao Liu, Xiaoying Lyu, Saroj K. Pradhan, Yiming Li, Xingfang Liu, Ralf Bauder, Tanja Heggli, Xiaying Wang, Michael Furian

**Affiliations:** 1 Swiss University of Traditional Chinese Medicine, Bad Zurzach, Aargau, Switzerland; 2 Shanghai University of Traditional Chinese Medicine, Shanghai, China; 3 Institute of Basic Research in Clinical Medicine, China Academy of Chinese Medical Sciences, Beijing, China; Endeavour College of Natural Health, AUSTRALIA

## Abstract

**Background:**

Traditional Chinese Medicine (TCM) is increasingly integrated into healthcare and insurance systems, therefore, it is essential to understand its current status and patients’ perspectives.

**Methods:**

This cross-sectional study was conducted from January 1^st^ to December 31^st^, 2023, across five TCM practices in Switzerland. All patients attending their sixth therapy session were invited to complete an electronically anonymized questionnaire covering patient demographics, treatment experiences, and satisfaction.

**Results:**

A total of 461 patients participated in the survey, with the majority being female (60.1%) and aged 50 years or older (57.4%). Among them, 54.0% reported multiple health conditions, with 32.9% having musculoskeletal disorders and 31.8% suffering from chronic pain as the main reasons for seeking therapy. Most patients received weekly TCM treatments (91.3%), with 50.7% also undergoing conventional therapies. Of the respondents, 50.0% reported full coverage for their TCM therapy costs. Access to TCM was primarily through personal recommendations (44.5%), and 92.2% of patients reported waiting less than 10 minutes before each therapy session. Acupuncture was the predominant treatment (95.7%), with 35.8% receiving additional dietary advice. The overall patient satisfaction rate stood at 96.5%, with 99.5% indicating their intention to continue receiving TCM treatments. Notably, patients with full insurance coverage for TCM treatment costs demonstrated significantly higher treatment satisfaction compared to those with no coverage (odds ratio = 2.42, 95% confidence interval [1.10 to 5.31], p = 0.028). By contrast, other evaluated medical factors did not show statistically significant associations with treatment satisfaction.

**Conclusion:**

This study revealed that women, patients aged over 50, and individuals with multiple health conditions, particularly musculoskeletal disorders and chronic pain, are more likely to seek regular integrated TCM treatment. Patients reported high levels of satisfaction with TCM, with treatment satisfaction significantly higher among those with full insurance coverage for TCM treatment costs compared to those without coverage. Future research should enroll a broader range of TCM practices and patient populations to enhance the generalizability of these findings and accurately evaluate how insurance coverage influences TCM satisfaction, thereby better addressing patient needs.

## Background

Traditional Chinese Medicine (TCM), a well-established system rooted in thousands of years of clinical practice and based on concepts such as yin-yang and qi, encompasses therapeutic modalities including acupuncture, cupping, herbal medicine, and life cultivation [1]. In Europe, TCM is classified under the broader category of Complementary and Alternative Medicine (CAM), which includes medical practices and products not typically part of conventional Western medicine [2]. The recognition of TCM in Europe has advanced notably, with TCM herbal drugs incorporated into the *European Pharmacopoeia* [3] and the inclusion of TCM diagnostic patterns in Chapter 26 of the International Classification of Diseases, 11^th^ Revision (ICD-11) [4,5]. A literature search in 2012 estimated at least 305,000 registered CAM providers in the European Union, acupuncture (n = 96,380) was the most widely therapeutic method for both medical (80,000) and non-medical (16,380) practitioners [6]. Research indicates that the use of CAM in Switzerland shares certain similarities with that in other European and Western countries, allowing for meaningful comparisons. However, notable differences also exist and should be taken into account [7,8].Thus, investigating the situation of TCM in Switzerland has significant relevance [9].

Since 2012, in Switzerland, TCM has been covered by basic health insurance when provided by certified physicians, and by supplementary insurance when administered by accredited therapists [10]. As a result, the use of Complementary Medicine (CM)—a subset of CAM referring to non-mainstream approaches used alongside conventional treatments—increased significantly from 24.7% in 2012 to 28.9% in 2017 (p < 0.001) [11]. Moreover, cross-sectional surveys conducted among representative samples of Swiss adults showed a steady rise in TCM use, from 6.6% in 2007 (including acupuncture and other TCM therapies) to 8.5% in 2017, a statistically significant increase [9,11].

In 2008, Michlig et al. conducted a prospective study in which patients reported significantly higher satisfaction after a 4-week follow-up when receiving TCM treatment from conventional physicians with additional TCM certification compared to conventional medicine therapy alone. However, there was no significant difference in complete recovery or marked symptom improvement [12]. Nevertheless, this study did not identify the TCM-related factors influencing treatment satisfaction. Since most TCM providers are not Western Medicine physicians, further research is warranted.

Although there are many studies on CAM from various databases, research on the real-world application of TCM and patient perspectives remains limited [13,14]. Therefore, this cross-sectional study investigates patient characteristics, medical factors, treatment modalities, and satisfaction among individuals undergoing TCM treatment in five practices during the first year of implementing a satisfaction questionnaire.

## Methods

### Study design

This cross-sectional study was conducted between January 1 and December 31, 2023, across five TCM practices in Switzerland (practices located in Bad Zurzach, Baden, Lenzburg, Wil, Zug). All participating practices adhered to standardized treatment protocols and employed certified TCM providers, ensuring a consistent quality of care across sites. Among the ten TCM providers surveyed in this study, four held bachelor’s degrees, two held master’s degrees, and four held doctoral degrees in TCM. Two TCM providers were licensed medical doctors. Overall, their average duration of TCM clinical practice was 22.7 ± 11.4 years, ranging from 10 to 40 years.

In agreement with the Swiss Human Research Act (HRA) and due to the assessment of anonymized data, this survey did not require ethical approval. This was confirmed by the local Ethics Committee Northwest and Central Switzerland (Req-2022–01098). The study was conducted according to the guidelines of the Declaration of Helsinki. The completion and findings of the individually completed questionnaires were not available to the TCM practice staff during the assessment period.

### Study population and eligibility

The survey was distributed to adult patients who had provided written informed consent, and to underage patients with consent provided by their parents or legal guardians. Eligible patients were German- or English-speaking patients who had completed six sessions of TCM treatment at one of the five participating practices, typically over a period of about three weeks. Patients were included in the survey regardless of whether they intended to continue or discontinue treatment beyond the sixth session. This timing was chosen to ensure that participants had received sufficient exposure to the treatment to provide a meaningful assessment. Patients who had attended fewer than six therapy sessions or lacked adequate reading or language proficiency were excluded.

### Data collection and management

The survey was created using EvaSys software [15] and completed by patients on a tablet after completing their sixth treatment session. An example of the questionnaire is provided in the Supporting Information (S1 File). It collected a wide range of information, including demographics (e.g., sex and age), medical factors (e.g., treatment season, self-reported health conditions, treatment regularity, concurrent treatment with conventional medicine, and insurance status and coverage), TCM treatment modalities, and aspects of patient satisfaction.

The questionnaire was developed collaboratively by certified TCM providers and academic researchers, drawing on existing instruments and expert recommendations. It was reviewed by multiple external consultants with prior collaboration experience with the World Health Organization (WHO) Traditional, Complementary and Integrative Medicine (TCI) Unit. The original version was in English and was translated into German using forward and backward translation by two independent bilingual translators. Discrepancies were resolved through consensus. To ensure clarity and feasibility, a three-month pilot study was conducted in 2022, during which a total of 151 patients completed the survey. Based on the feedback from these patients, TCM providers, and the administration, the final questionnaire was developed. The survey was administered electronically with built-in validation features (e.g., required fields) to minimize data entry errors. Anonymous participation was employed to encourage honest responses and reduce social desirability bias.

As the study focused on descriptive analyses of observed patient characteristics and satisfaction patterns rather than measurement of latent constructs, formal psychometric validation (e.g., reliability or factor analysis) was not prioritized. Future research will consider systematic validation of the instrument’s measurement properties.

To maintain data quality and transparency, all statistical analyses were based on valid responses per item. Missing data were not imputed, consistent with the study’s aim to reflect real-world observational data rather than conduct complete-case analyses. Variation in response rates across items primarily resulted from the optional nature of certain questions and minor questionnaire adjustments during the study period.

### Statistical analysis

The reporting of the findings of this study follows the Strengthening the Reporting of Observational Studies in Epidemiology (STROBE) guidelines [16]. The data are summarized as numbers and proportions. Missing data of questionnaire items were not imputed and data analysis was conducted in all patients submitting a questionnaire. Comparisons between proportions were conducted with χ² tests. Ordered logistic regression analysis examined the relationship between treatment satisfaction, patient demographics, and medical factors. A two-sided p < 0.05 was considered to reflect statistical significance. All statistical analyses were conducted with the software R, version 4.0.5 (R Foundation for Statistical Computing in Vienna, Austria).

## Results

### Study participation

Out of 1,095 patients who initiated a new TCM therapy in 2023, 473 received a sixth session, and 461 completed and returned anonymous satisfaction surveys, while 12 individuals declined to participate. Overall, 94.6% of all survey items were answered, resulting in missing data in 5.4%.

### Patient and TCM practice characteristics

In the study, 277 (60.1%) were women, significantly more than men (p < 0.001). Patients aged ≥50 years outnumbered those aged <50 years (p = 0.002). The median age category was 50–60 years, and the average age of male patients was higher than that of female patients (Table 1, eFig 1). In terms of treatment regularity, patients receiving weekly treatments (91.3%) significantly outnumbered those receiving treatments irregularly (p < 0.001). Half of all respondents reported concurrent treatment with conventional medicine. There was a significant difference in insurance coverage for TCM treatment costs (p < 0.001); 50% reported that they could cover the TCM treatment expenses fully, 45% partially, and 5% not at all. There were statistically significant differences in patient enrollment across the four seasons, with winter showing the lowest proportion of patients (19.5%) (Table 1).

**Table 1:** Characteristics of TCM practices and patients seeking TCM therapy.

	N (%)	Odds ratio^1^	95% CI	p-value
**Sex (444 responses)**				
Female	277 (60.1)	1 [Reference]		
Male	167 (36.2)	1.09	0.75 to 1.58	0.643
**Age (444 responses)**				
< 50 years	189 (42.6)	1 [Reference]		
≥ 50 years	255 (57.4)	1.00	0.70 to 1.43	0.994
**Treatment season (461 responses)**				
Spring (Mar-May)	124 (26.9)	1 [Reference]		
Summer (Jun – Aug)	103 (22.3)	1.05	0.64 to 1.72	0.854
Autumn (Sep – Nov)	144 (31.2)	1.25	0.79 to 1.98	0.341
Winter (Dec – Feb)	90 (19.5)	1.25	0.74 to 2.13	0.403
**Condition Count (461 responses)**				
Single health condition	212 (46.0)	1 [Reference]		
Multiple health conditions	249 (54.0)	1.16	0.81 to 1.66	0.427
Treatment regularity* (229 responses)				
Weekly	209 (91.3)	1 [Reference]		
Irregular	20 (8.7)	0.83	0.36 to 1.95	0.674
Concurrent treatment with conventional medicine* (219 responses)				
Yes	111(50.7)	1 [Reference]		
No	108 (49.3)	0.75	0.45 to 1.26	0.276
**Insurance Status (441 responses)**				
Private payments	18 (4.1)	1 [Reference]		
Supplementary health insurance	278 (63.0)	1.08	0.44 to 2.65	0.868
Basic health insurance only	145 (32.9)	1.00	0.40 to 2.52	0.999
**Insurance coverage for TCM treatment costs (436 responses)**				
None	22 (5.0)	1 [Reference]		
Yes, partially	196 (45.0)	1.42	0.65 to 3.11	0.383
Yes, in full	218 (50.0)	2.42	1.10 to 5.31	0.028

Values presented as numbers and proportions. ^1^Odds ratio = odds ratio of positive treatment satisfaction choice; CI = confidence interval; *This item was implemented in June 2023. Therefore, fewer responses were recorded until December 2023. TCM, traditional Chinese medicine.

**Fig 1:**
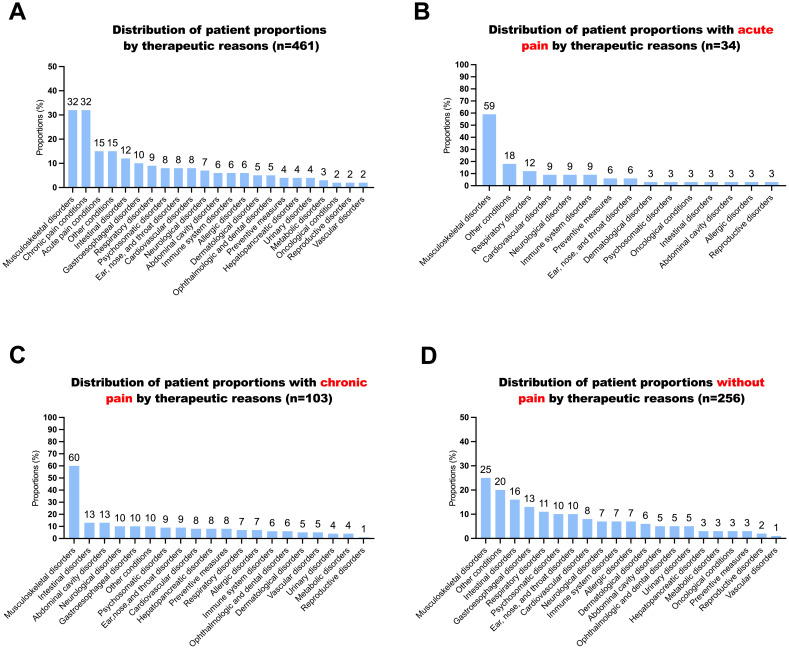
Distribution of patient proportions by therapeutic reasons. Panel A shows the proportion of overall therapeutic reasons; Panel B shows the proportion of therapeutic reasons under acute pain; Panel C shows the proportion of therapeutic reasons under chronic pain; Panel D shows the proportion of therapeutic reasons without pain.

### Medical factors

The survey listed 23 possible self-reported health conditions for seeking TCM treatment. Each patient reported an average of 2.0 ± 1.5 different health conditions, ranging from 1 to 9. Patients with multiple conditions (54.0%) outnumbered those with a single condition (46.0%), although the difference was not statistically significant. Among all patients, the most reported reasons for seeking TCM treatment were musculoskeletal disorders (33%) and chronic pain conditions (32%) (Fig 1, Panel A). Both acute and chronic pain conditions were closely linked with musculoskeletal disorders (Fig 1, Panels B to D).

### TCM treatment modalities

Patients were primarily introduced to TCM through personal recommendations (45%) and the Internet (23%). Most patients reported minimal average waiting times before each therapy session, with over 50% indicating no waiting time at all. The patients reported an average of 1.5 ± 0.4 different treatment therapies and 1.3 ± 0.1 different lifestyle modification recommendations. Acupuncture was the most used therapy in more than 95% of cases. Many patients received lifestyle advice, particularly on diet. Overall, 99.5% of patients were positive about continuing TCM treatment (Table 2).

**Table 2:** TCM treatment modalities.

Parameters	n (%)
**How did you first learn about TCM? (461 responses)**	
Through recommendation	205 (44.5)
Through the internet	104 (22.6)
Through prior treatment	85 (18.4)
Through medical referral	74 (16.0)
Through an event	30 (6.5)
Through advertisement	16 (3.5)
Through other reasons	13 (2.8)
Through social media	2 (0.4)
**Average waiting time over the first six therapy sessions (448 responses)**	
No Waiting Time	243 (54.2)
Up to 5 Min	128 (28.6)
Up to 10 Min	42 (9.4)
Up to 20 Min	18 (4.0)
Up to 30 Min	13 (2.9)
More than 30 Min	4 (0.9)
**Applied TCM treatment therapy (461 responses)**	
Acupuncture	441 (95.7)
Cupping	135 (29.3)
Acupressure and massage	65 (14.1)
Chinese herbal medicine	19 (4.1)
Moxibustion	5 (1.1)
Other Therapy	4 (0.9)
**Additional advice on lifestyle modification (461 responses)**	
Diet	165 (35.8)
Other recommendations	123 (26.7)
Sleep hygiene	94 (20.4)
Physical activity	91 (19.7)
No specific recommendations	90 (19.5)
Clothing and thermal regulation	60 (13.0)
Continue of TCM treatment* (227 responses)	
Definitely yes	166 (73.1)
Rather yes	60 (26.4)
Rather no	1 (0.4)
Definitely no	0 (0.0)

n = number of responses; * This item was implemented in June 2023. Therefore, fewer responses were recorded until December 2023. TCM, traditional Chinese medicine.

### Patient-reported satisfaction

**Fig 2:**
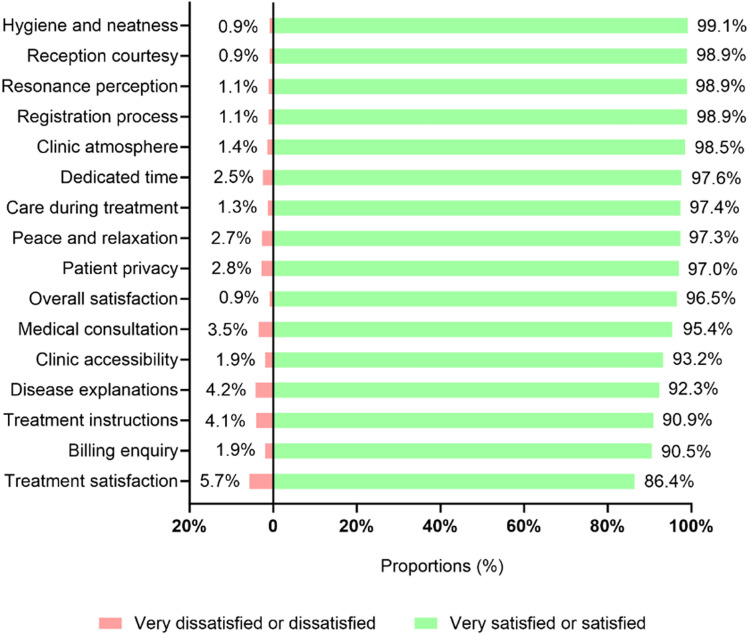
Patients’ satisfaction with various TCM practices and treatment aspects. TCM, traditional Chinese medicine.

Overall satisfaction of all respondents was 96.5% and similar across multiple domains, including treatment satisfaction, medical environment, administration, and TCM providers (Table 3, Fig 2). Treatment satisfaction was reported by 86.3% of respondents, with 52.3% indicating they were “very satisfied” and 34.0% “satisfied,” while 5.7% were dissatisfied and 8.0% unable to evaluate. Other domains, such as billing inquiry, medical consultation, disease explanation, and treatment instruction, received “very satisfied” ratings of 65.9%, 69.4%, 60.4%, and 60.7%, respectively (Table 3, Fig 2).

**Table 3:** Satisfaction with various aspects of the TCM practices and treatment.

	Very Satisfied	Satisfied	Dissatisfied	Very Dissatisfied	Unable to evaluate
**Overall satisfaction (461 responses)**	NA	96.5%	0.9%	NA	2.6%
**Treatment satisfaction (461 responses)**	52.3%	34.0%	5.0%	0.7%	8.0%
**Medical environment**					
Hygiene and neatness (448 responses)	93.8%	5.4%	0.2%	0.7%	0.0%
Clinic atmosphere (444 responses)	79.1%	19.4%	0.9%	0.5%	0.2%
Peace and relaxation (449 responses)	81.5%	15.8%	2.0%	0.7%	0.0%
Patient privacy (438 responses)	75.1%	21.9%	2.1%	0.7%	0.2%
**Administration**					
Registration process (448 responses)	90.9%	8.0%	0.0%	1.1%	0.0%
Reception courtesy (443 responses)	93.9%	5.0%	0.0%	0.9%	0.2%
Billing inquiry (431 responses)	65.9%	24.6%	1.4%	0.5%	7.7%
Clinic accessibility (428 responses)	78.7%	14.5%	1.2%	0.7%	4.9%
**TCM provider**					
Resonance perception (447 responses)	87.5%	11.4%	0.2%	0.9%	0.0%
Care during treatment (460 responses)	86.1%	11.3%	0.4%	0.9%	1.3%
Dedicated time (444 responses)	82.7%	14.9%	1.8%	0.7%	0.0%
Medical consultation (435 responses)	69.4%	26.0%	3.0%	0.5%	1.1%
Disease explanation (432 responses)	60.4%	31.9%	3.7%	0.5%	3.5%
Treatment instruction (417 responses)	60.7%	30.2%	3.4%	0.7%	5.0%

Values represent proportions. TCM, traditional Chinese medicine; NA, not assessed.

In univariate ordered logistic regression analyses, full insurance coverage for TCM treatment costs was positively associated with higher treatment satisfaction (Table 1). Other variables did not show relevant associations with treatment satisfaction (Table 1).

## Discussion

Traditional Chinese Medicine (TCM) has gained increasing attention as a complementary approach to conventional healthcare, yet detailed insights into the characteristics of patients seeking TCM and their treatment experiences remain limited. In this cross-sectional study conducted in five Swiss TCM practices in 2023, we aimed to explore patient demographics, medical factors, treatment modalities and satisfaction. Our findings revealed that patients presenting for TCM therapy exhibit a broad spectrum of underlying health conditions and report high satisfaction after the sixth therapy session.

The demographic characteristics, including a higher proportion of women and older adults utilizing TCM, align with global patterns where women and elderly individuals tend to use complementary medicine more frequently(eFig 1), possibly related to their higher prevalence of chronic diseases [9,17–19]. TCM’s holistic approach, addressing both physical and emotional health aspects, potentially complements conventional medical treatments and meets patients’ diverse needs [20].

The most commonly treated health conditions, as self-reported by patients, were musculoskeletal disorders, pain, and gastrointestinal conditions, which are consistent with areas where TCM, especially acupuncture, has demonstrated evidence-based efficacy [21]. Clinical guidelines recommend non-drug interventions such as acupuncture and multidisciplinary rehabilitation for conditions like low back pain [22]. Furthermore, acupuncture has demonstrated positive outcomes in treating gastroesophageal reflux disease, ulcerative colitis, and Crohn’s disease [23–25]. These findings underscore TCM’s potential role within modern multidisciplinary healthcare, highlighting the need for further prospective clinical research and interdisciplinary collaboration to better define its effectiveness and applications [26].

No correlation was found between treatment satisfaction and factors such as patient age, sex, treatment season. Interestingly, the study also found that patients with full insurance reimbursement reported greater satisfaction than those with no coverage, reflecting the critical impact of economic accessibility on patient experience and treatment adherence [27]. Financial burdens may deter patients from continuing therapy despite perceived benefits, indicating that improving insurance coverage could enhance adherence and satisfaction, thereby potentially improving clinical outcomes.

Acupuncture was the predominant treatment modality used in this study, received by 95.7% of patients, consistent with national survey data and clinical practice norms [11,28]. Its well-documented efficacy in musculoskeletal pain explains its widespread use [21], while TCM’s integrative approach often combines multiple modalities to restore body balance, which may further contribute to perceived benefits.

Patient satisfaction is a complex, multidimensional outcome influenced by the medical environment, administrative processes, treatment efficacy, and communication [29]. Although patients generally valued the medical environment and administration, gaps were noted in the transparency of billing and insurance coverage information. Providing clear, upfront explanations of treatment costs and reimbursement policies may improve adherence and satisfaction, particularly among patients with limited insurance coverage. Moreover, dedicating sufficient time for consultations to explain diagnoses, treatment rationale, and instructions could enhance patient understanding and engagement. Variations in satisfaction across disease types warrant further exploration through prospective clinical trials to elucidate underlying factors.

This study has several limitations. First, the reliance on self-reported questionnaires may introduce reporting bias, as patients might provide higher satisfaction ratings due to social desirability. Second, surveying patients at the sixth treatment session helps ensure sufficient treatment exposure for meaningful satisfaction assessment, but may still introduce selection bias, as dissatisfied patients may have discontinued treatment earlier and were therefore not captured. Moreover, the survey likely missed acute illnesses, which are commonly treated with less than six therapy sessions. The lack of classification of patient health conditions according to the International Classification of Diseases (ICD-10), combined with the fact that health conditions were self-reported by patients, limits the precise assessment of disease status and relevant influencing factors. However, those health conditions represent real-world complaints of patients seeking a TCM therapy. Many of those patients and health conditions are not diagnosed or classified by ICD-10. Finally, as the study was conducted solely in five Swiss TCM practices, the external validity of findings should be confirmed through similar research in other cultural and healthcare contexts.

Future research should include prospective studies to validate the impact of insurance coverage on long-term adherence and integrate subjective satisfaction measures with objective health outcomes, such as pain scores and functional improvements, to comprehensively evaluate TCM treatment effectiveness.

## Conclusion

This cross-sectional study conducted in five Swiss TCM practices showed that patients seeking TCM therapy present with diverse health conditions, primarily musculoskeletal disorders, and generally report high treatment satisfaction after six treatment sessions. Acupuncture combined with lifestyle recommendations was the main treatment approach. Those findings indicate that acupuncture could be considered when patient satisfaction for Western Medicine becomes questionable. The study found that insurance coverage significantly influences treatment satisfaction, highlighting the important role of economic accessibility in patient adherence and experience. This association between treatment satisfaction and financial coverage of TCM therapies implies that the overall situation remains challenging for patients.

## Supporting information

S1 File Questionnaire. (PDF)

S2 File eFig 1: Age distribution in female and male returning a satisfaction questionnaire; STROBE Checklist for Reporting Cross-sectional Studies. (DOCX)

S1 Data 2023 Satisfaction Survey Summary. (XLSX)
